# PREVENT: Towards the prevention of intracranial vessel perforations during mechanical thrombectomy – protocol for a multicenter registry study

**DOI:** 10.3389/fneur.2025.1680379

**Published:** 2026-01-23

**Authors:** Victor Schulze-Zachau, Nikki Rommers, Hannah Muenger, Gundeep Kaur Singh, Mira Katan, Luzia Balmer, Sofie Psychogios, Alex Brehm, Urs Fischer, Marios-Nikos Psychogios

**Affiliations:** 1 Department of Diagnostic & Interventional Neuroradiology, Radiology & Nuclear Medicine Clinic, University Hospital Basel, Basel, Switzerland; 2 Department of Clinical Research, University of Basel, Basel, Switzerland; 3 Neurology Clinic, University Hospital Basel, Basel, Switzerland; 4 Neurology Clinic, University Hospital Bern, Bern, Switzerland

**Keywords:** stroke, thrombectomy, complication, intracranial hemorrhage, multicenter

## Abstract

**Rationale:**

The increasing use of thrombectomy for acute ischemic stroke highlights the need to better understand its complications. Vessel perforation leading to active intracranial bleeding is a severe complication associated with a poor outcome and a mortality rate of approximately 50%. The etiology and risk factors for this severe complication remain largely unknown, and there is limited evidence to guide decision-making regarding endovascular hemostatic strategies and the continuation of thrombectomy after perforation.

**Design:**

The Perforation EVents during ENdovascular Therapy for acute ischemic stroke (PREVENT) Registry is an international, multicenter, prospective, and retrospective study, collecting data on vessel perforations during thrombectomy. The registry aims to include 500 cases of vessel perforation and 500 matched controls without perforation. The collected data will consist of both tabulated data and periprocedural imaging, which is to be analyzed centrally by an imaging core laboratory. Data will be analyzed using a case–control method, employing both univariate and multivariate statistical analyses.

**Objectives:**

The primary objectives of this study are to (1) identify risk factors for vessel perforation, (2) explore the underlying pathophysiology, (3) develop a classification system for vessel perforations, (4) compare different hemostatic treatment strategies, (5) evaluate the impact of continuing versus aborting thrombectomy after perforation, and (6) propose a safety-optimized thrombectomy technique.

**Discussion:**

Given the frequency and hyperacute nature of vessel perforations, a prospective randomized study is not feasible. This large, international registry provides a robust approach to collecting real-world evidence, reducing bias through multicenter data collection and case matching. The findings may help improve clinical decision-making and enhance patient safety during thrombectomy procedures.

**Clinical trial registration:**

ClinicalTrials.gov, identifier NCT06394180.

## Introduction

Stroke is the second leading cause of death and the leading cause of disability, both globally and in Europe (1, 2). Thrombectomy has revolutionized the treatment of acute ischemic stroke, following evidence from five randomized controlled trials demonstrating a substantial benefit in patients with large-vessel occlusion of the anterior circulation (3). Since then, the indications for thrombectomy have continued to expand (4), including patients with posterior circulation occlusions (5, 6), selected patients presenting late after symptom onset (7–9), and patients with large infarcts (10, 11). Patients with medium or distal vessel occlusions (MDVOs) are also increasingly being considered for thrombectomy (4, 12, 13). Two recent randomized controlled trials did not demonstrate the superiority of MDVO thrombectomy over best medical treatment alone (14, 15). One trial reported a higher number of symptomatic intracranial hemorrhages (sICHs) and a higher mortality in the thrombectomy group (14). A third trial was stopped early for futility and also reported a higher rate of sICH in the thrombectomy group compared to the best medical management group (16).

These findings might point towards a different aspect of thrombectomy: despite its effectiveness, the procedure is not without the risk of periprocedural complications. Intracranial vessel perforation with subsequent arterial bleeding occurs in 1–6% of thrombectomies (17–20). This complication is typically a critical event, with permanent consequences (21, 22) and a mortality rate of approximately 50% (17, 19, 23). Given the rapidly increasing number of thrombectomies, an increase in the incidence of periprocedural perforations is to be expected. This is particularly relevant for MDVO thrombectomy, as the incidence of vessel perforations has been reported to be twice that of LVO thrombectomy (17).

The available data on vessel perforations during thrombectomy are limited (21). It is unknown why some patients experience this complication, while the majority are spared. Similarly, the underlying pathophysiological process that leads to vessel wall damage is not understood. There is uncertainty about which patients benefit from endovascular hemostatic treatment to achieve cessation of active bleeding and whether temporary vessel occlusion (e.g., using an intracranial balloon catheter or by deploying coils without detachment) or permanent vessel occlusion (e.g., using liquid embolic agents or permanent coiling) is more appropriate. In addition, limited data are available on whether thrombectomy should continue after a perforation occurs or whether the procedure should be aborted. Given the low frequency of this complication and its hyperacute nature, randomized prospective trials are unlikely to be feasible.

## Methods and analysis

### Design

The Perforation EVents during ENdovascular Therapy for acute ischemic stroke (PREVENT) Registry is an international, multicenter, prospective, and retrospective observational study collecting data on vessel perforations during thrombectomy.

Data collection includes the following:

- Patient baseline data (e.g., age, sex, pre-stroke modified Rankin Scale (mRS), pre-stroke anticoagulation or platelet therapy, and others).
- Data on the initial ischemic event (e.g., NIHSS at admission, number and location of vessel occlusions, TOAST classification [trial of ORG 10172 in Acute Stroke Treatment], imaging features related to clot composition, intracranial atherosclerotic disease (ICAD) or primary intracranial dissection, non-interventional stroke management, and other relevant factors).
- Details on circumstances and technique of the thrombectomy (e.g., anesthesiological management, type of aortic arch, cervical stenosis, thrombectomy technique, design and dimensions of the thrombectomy devices, operator experience, and others).
- Hemostatic therapy (e.g., non-endovascular hemostatic measures such as lowering of the systemic blood pressure or medication with coagulation effects, endovascular hemostatic measures including timing, exact mechanism, effectiveness, and other relevant factors).
- Outcome measures (e.g., functional outcomes including mRS at 90 days, and imaging outcomes including recanalization status, volume of intracranial hemorrhage, and other relevant factors).

The collected data are stored safely in a REDCap database. Periprocedural imaging and the first postinterventional CT or MRI are obtained for the extraction of quantifiable imaging parameters, exploration of patterns, and quantification of the volume of extravasated blood and contrast agents. Imaging data are stored in a safe, password-protected Picture Archiving and Communication System (PACS) at Basel University Hospital, Basel, Switzerland. The duration of active intracranial bleeding is derived from the time stamps of the digital subtraction angiography series during thrombectomy displaying contrast extravasation. The collateral status will be derived from the first intraprocedural angiographic run. The flow rate of extravasation (i.e., the volume of extravasation per second) will be classified by the imaging core laboratory based on the first angiographic run that demonstrates extravasation. This will be used as a surrogate for perforation magnitude. A semi-quantitative assessment of the flow rate of extravasation will be sought, if technically feasible. Imaging evaluation will be carried out by a central imaging core laboratory to ensure a consistent interpretation.

PREVENT exclusively consists of data and imaging that have initially been collected for clinical purposes of patient care. As an observational study, PREVENT does not contain a randomization.

PREVENT aims to answer the following questions:

1. Which risk factors are associated with the occurrence of vessel perforations during thrombectomy?
2. Is it possible to identify distinct mechanisms of vessel perforation? Is it possible to describe pathophysiological phenomena associated with vessel perforation?
3. Are there groups of perforations that can be distinguished in terms of risk profiles and the need for urgent complication management, on the basis of which a classification system can be created? If so, can a pathophysiological explanation be found for the differences between these groups?
4. In patients with ongoing contrast extravasation, which individuals should receive endovascular therapy to achieve hemostasis? How do specific hemostatic strategies compare to others regarding efficacy and safety?
5. In patients in whom ongoing contrast extravasation has stopped, should thrombectomy be continued, or should the procedure be aborted?
6. Based on the insights gained from PREVENT, is it possible to design a safety-optimized thrombectomy technique that may be used if patient-related risk factors imply an increased risk of vessel perforation?

**Figure 1:**
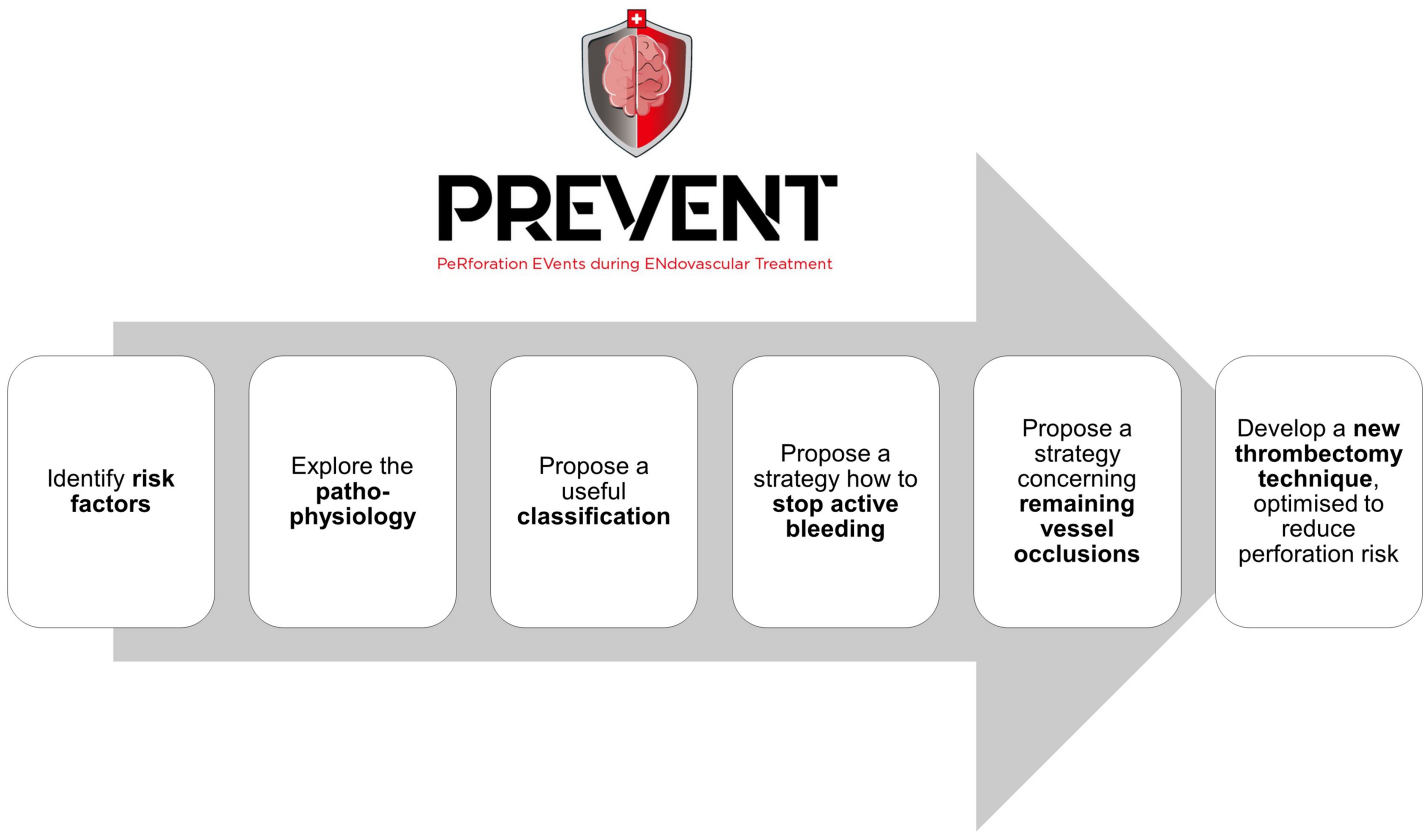
Aims of the PREVENT registry. PREVENT, Perforation EVents during ENdovascular Therapy for acute ischemic stroke.

Figure 1 shows the aims of the PREVENT registry.

### Selection/treatment of subjects

Adult patients (≥18 years) who underwent thrombectomy for acute ischemic stroke on or after 1 January 2015 and experienced a periprocedural vessel perforation will be included. Vessel perforation is defined as contrast extravasation in at least one angiography series. Patients will be excluded if intracranial hemorrhage was present on pre-interventional imaging, if vessel wall damage was due to intracranial dissection without active contrast extravasation, if vessel perforation was due to the rupture of a pre-existing intracranial aneurysm (i.e., extravasation originates from an aneurysmal outpouching or dilation of an intracranial artery on the first run that demonstrates extravasation), or if vessel perforation led to the development of an arteriovenous fistula (e.g., carotid-cavernous fistula), but not to subarachnoid hemorrhage.

To evaluate risk factors that predispose patients to perforations, PREVENT is designed as a case–control registry. For the inclusion of each patient who experiences a perforation during thrombectomy, one matched patient of the same sex and similar age, with acute ischemic stroke due to vessel occlusion in the same vascular segment and treated at the same center, but without perforation, will be included.

### Data analysis

Summary tables for patient demographics as well as baseline and intervention characteristics will be provided. Further statistical analysis will be tailored for each of the study’s aims. The methods per aim are presented below, following the same order:

1. To identify risk factors associated with vessel perforations, we will, in the first stage, make comparisons between cases with and without perforation during thrombectomy for predefined risk factors. Statistical analyses will include paired samples *t*-test, Wilcoxon test, and chi-squared test or Fisher’s exact test. Furthermore, multivariable logistic regression models can be used to identify the association between multiple variables and the occurrence of vessel perforations.
2. An exploratory analysis of the interventional image material in the central image analysis laboratory is planned. After reviewing the image material of approximately 20% of patients with explorative intention, imaging criteria will be defined with regard to possible mechanisms of vessel wall injury and distinct pathophysiological phenomena. These criteria will be used to classify the remaining 80% of patients. Due to the exploratory nature, no definitive statistical evaluation strategy can yet be determined.
3. For this purpose, data-driven K-nearest neighbor clustering will be applied to identify different groups of similar patient characteristics. We will consider 2–4 clusters, and the distribution of the clusters will be inspected visually. After the identification of the clusters, we will compare the pathophysiological characteristics between the clusters.
4. Patients with perforation will be divided into two groups: patients who received interventional hemostatic therapy after the perforation occurred—e.g., temporary intracranial balloon occlusion or permanent vessel occlusion with liquid embolization or coils—and patients who did not receive hemostatic therapy. Propensity score matching or inverse probability of treatment weighting will be used to control for the location of perforation and the flow rate of extravasation. The two groups will be compared with each other to identify differences in the duration of extravasation and in clinical outcomes. The appropriate statistical model will be used for each of the clinical outcomes (e.g., logistic regression model for binary outcomes and ordinal regression model for ordinal outcomes).
5. Patients with perforation will be divided into two groups: patients in whom thrombectomy was continued after the perforation occurred and patients in whom the procedure was aborted. Propensity score matching or inverse probability of treatment weighting will be used to control for the amount of extravasation, stroke severity, occlusion location, collateral status, and other clinical outcome predictors. The two groups will be compared with each other to identify differences in clinical outcomes.
6. The new insights on risk factors, mechanisms, and pathophysiology of vessel perforation will be gathered. An expert panel will discuss potential modifications to current thrombectomy workflows to reduce the risk of periprocedural perforation, both in response to specific patient-related risk factors (e.g., occlusion location and vessel tortuosity) and in general. No definitive statistical evaluation strategy can yet be determined.

## Discussion

Randomized controlled trials have established the efficacy of thrombectomy, providing a strong foundation for guideline recommendations (3, 5, 6, 8–11). However, as materials and interventional strategies continue to evolve, thrombectomy techniques are constantly being refined. Enhancing the safety of thrombectomy presents a unique challenge: prospective trials are typically not designed to detect complications that occur at low frequencies. However, these complications can be severe, often carrying profound or even fatal consequences for affected patients (17–19, 21, 23). Therefore, efforts to improve thrombectomy should focus not only on optimizing reperfusion but also on preventing complications whenever possible and enhancing complication management strategies. This becomes even more crucial given the expanding indications for thrombectomy. The net benefit of thrombectomy is presumed to be lower in patients with low National Institutes of Health Stroke Scale (NIHSS) scores at presentation or those with medium or distal vessel occlusions (MDVOs) than in the HERMES meta-analysis cohort. While minimizing adverse events is always important, the impact of severe complications may be even more pronounced in these patient populations since the natural course of disease is less severe. Consequently, improving procedural safety could help extend thrombectomy indications to patients who might otherwise be excluded due to safety concerns.

By focusing exclusively on vessel perforations during thrombectomy, the PREVENT registry represents a dedicated effort to target a specific safety aspect of thrombectomy. Unlike prospective randomized trials, which would be unfeasible due to the rarity and acute nature of this complication, PREVENT allows for the collection of a large, real-world dataset across multiple institutions. The matched case–control approach enables comparisons between patients with and without perforation under otherwise similar conditions. This may help to identify characteristics of the patient (e.g., neurovascular risk factors), the procedure setting (e.g., anesthesiologic strategy), or the devices (e.g., dimensions in relation to vessel diameters) that might be associated with increased risks. Additionally, the centralized imaging analysis ensures a standardized interpretation of angiographic and postprocedural imaging, allowing for the identification of distinct pathophysiological patterns of vessel perforation. A comparison between different hemostatic strategies (e.g., medical management only, temporary vessel occlusion, and permanent vessel occlusion) may provide insights into their effectiveness. By leveraging data that have been previously collected in routine clinical practice, PREVENT circumvents ethical and logistical barriers inherent in interventional trials while still providing robust insights into the mechanisms, management, and potential prevention of vessel perforations in thrombectomy.

## Author’s note

The study results will be submitted for presentation at large international scientific congresses and for publication in peer-reviewed scientific journals.
